# Supplementary material for: Acetyl-CoA carboxylase 1 and 2 inhibition ameliorates steatosis and hepatic fibrosis in a MC4R knockout murine model of nonalcoholic steatohepatitis
Source: PLoS One. 2020 Jan 28;15(1):e0228212. doi: 10.1371/journal.pone.0228212 (PMC6986730; doi:10.1371/journal.pone.0228212)
Supplement: S1 Fig — (PPTX) [file pone.0228212.s001.pptx]

## Slide 1
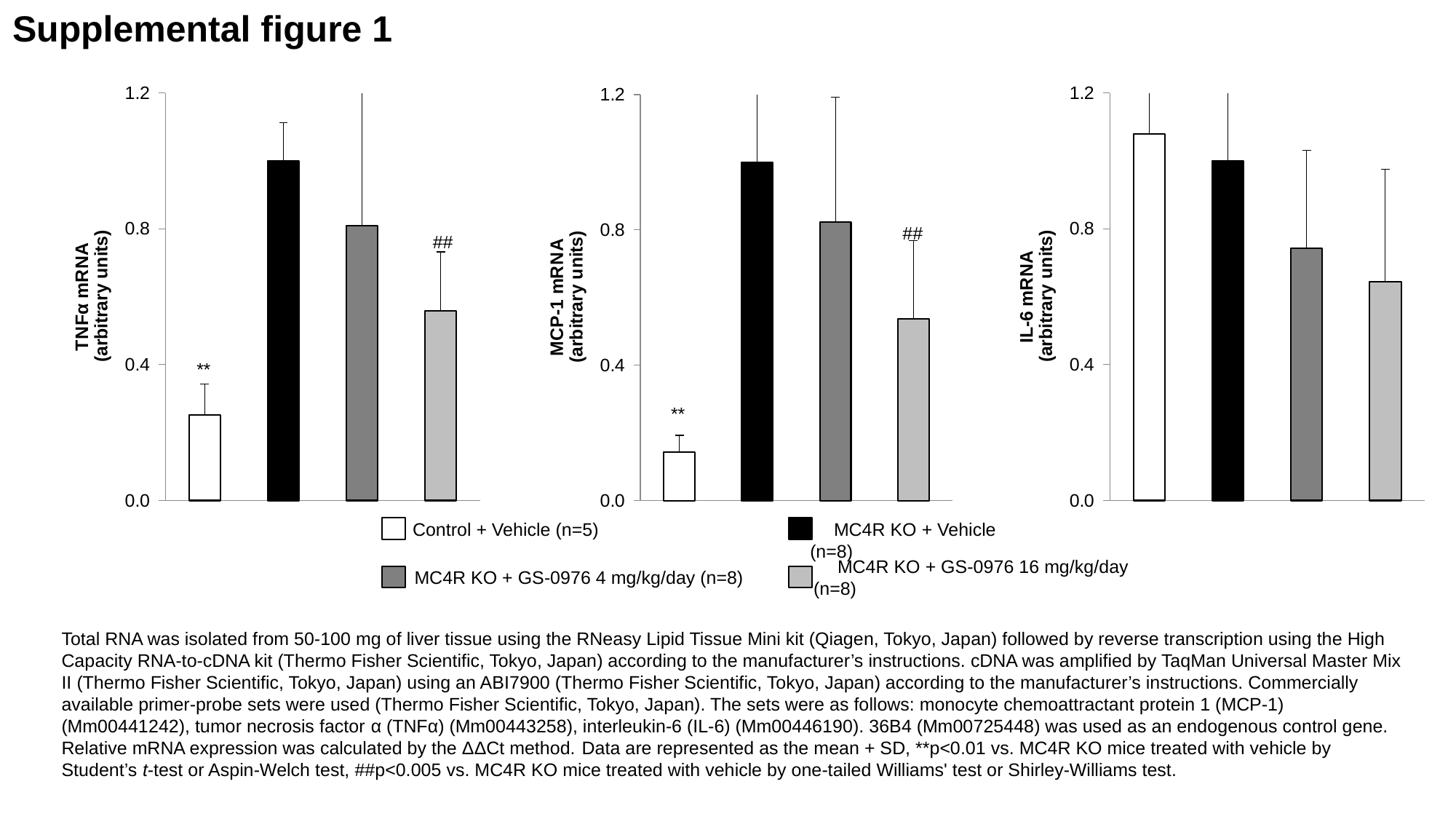

Supplemental figure 1
### Chart
| Category | |
|---|---|
| | 0.2522008580083599 |
| | 0.9999999999999999 |
| 2BID | 0.8090881842251317 |
| 8BID | 0.5581469095595039 |
### Chart
| Category | |
|---|---|
| | 0.14409784431086073 |
| | 1.0 |
| 2BID | 0.8230665485675736 |
| 8BID | 0.5366703077659873 |
### Chart
| Category | IL-6 |
|---|---|
| | 1.078714751264536 |
| | 1.0 |
| 2BID | 0.7425369171363434 |
| 8BID | 0.6443230704367392 | Control + Vehicle (n=5)
MC4R KO + Vehicle (n=8)
MC4R KO + GS-0976 16 mg/kg/day (n=8)
MC4R KO + GS-0976 4 mg/kg/day (n=8)
Total RNA was isolated from 50-100 mg of liver tissue using the RNeasy Lipid Tissue Mini kit (Qiagen, Tokyo, Japan) followed by reverse transcription using the High Capacity RNA-to-cDNA kit (Thermo Fisher Scientific, Tokyo, Japan) according to the manufacturer’s instructions. cDNA was amplified by TaqMan Universal Master Mix II (Thermo Fisher Scientific, Tokyo, Japan) using an ABI7900 (Thermo Fisher Scientific, Tokyo, Japan) according to the manufacturer’s instructions. Commercially available primer-probe sets were used (Thermo Fisher Scientific, Tokyo, Japan). The sets were as follows: monocyte chemoattractant protein 1 (MCP-1) (Mm00441242), tumor necrosis factor α (TNFα) (Mm00443258), interleukin-6 (IL-6) (Mm00446190). 36B4 (Mm00725448) was used as an endogenous control gene. Relative mRNA expression was calculated by the ΔΔCt method. Data are represented as the mean + SD, **p<0.01 vs. MC4R KO mice treated with vehicle by Student’s t-test or Aspin-Welch test, ##p<0.005 vs. MC4R KO mice treated with vehicle by one-tailed Williams' test or Shirley-Williams test.
