# Supplementary material for: Acetyl-CoA carboxylase 1 and 2 inhibition ameliorates steatosis and hepatic fibrosis in a MC4R knockout murine model of nonalcoholic steatohepatitis
Source: PLoS One. 2020 Jan 28;15(1):e0228212. doi: 10.1371/journal.pone.0228212 (PMC6986730; doi:10.1371/journal.pone.0228212)
Supplement: S1 Table — (PPTX) [file pone.0228212.s002.pptx]

## Slide 1
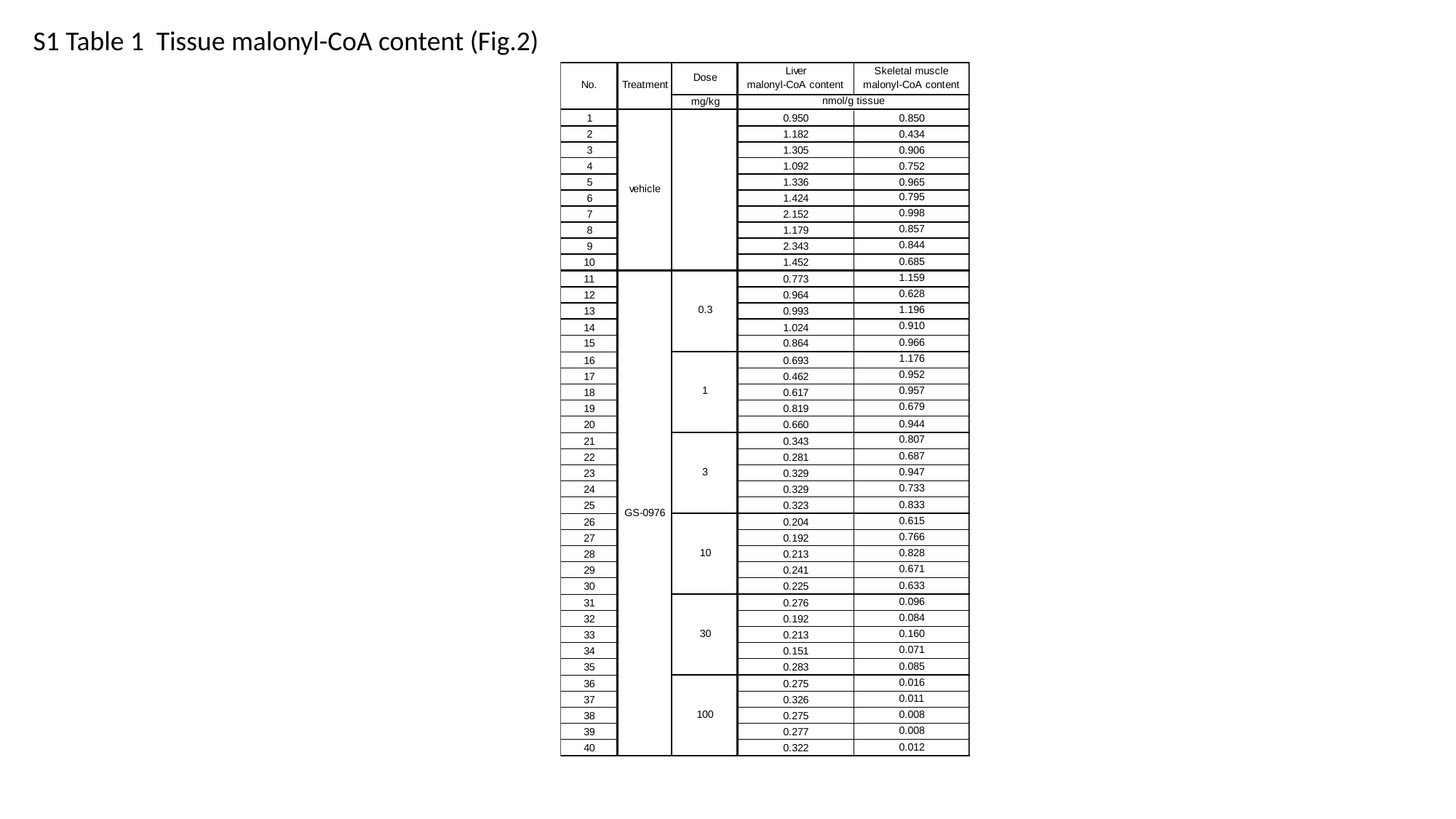

S1 Table 1 Tissue malonyl-CoA content (Fig.2)

## Slide 2
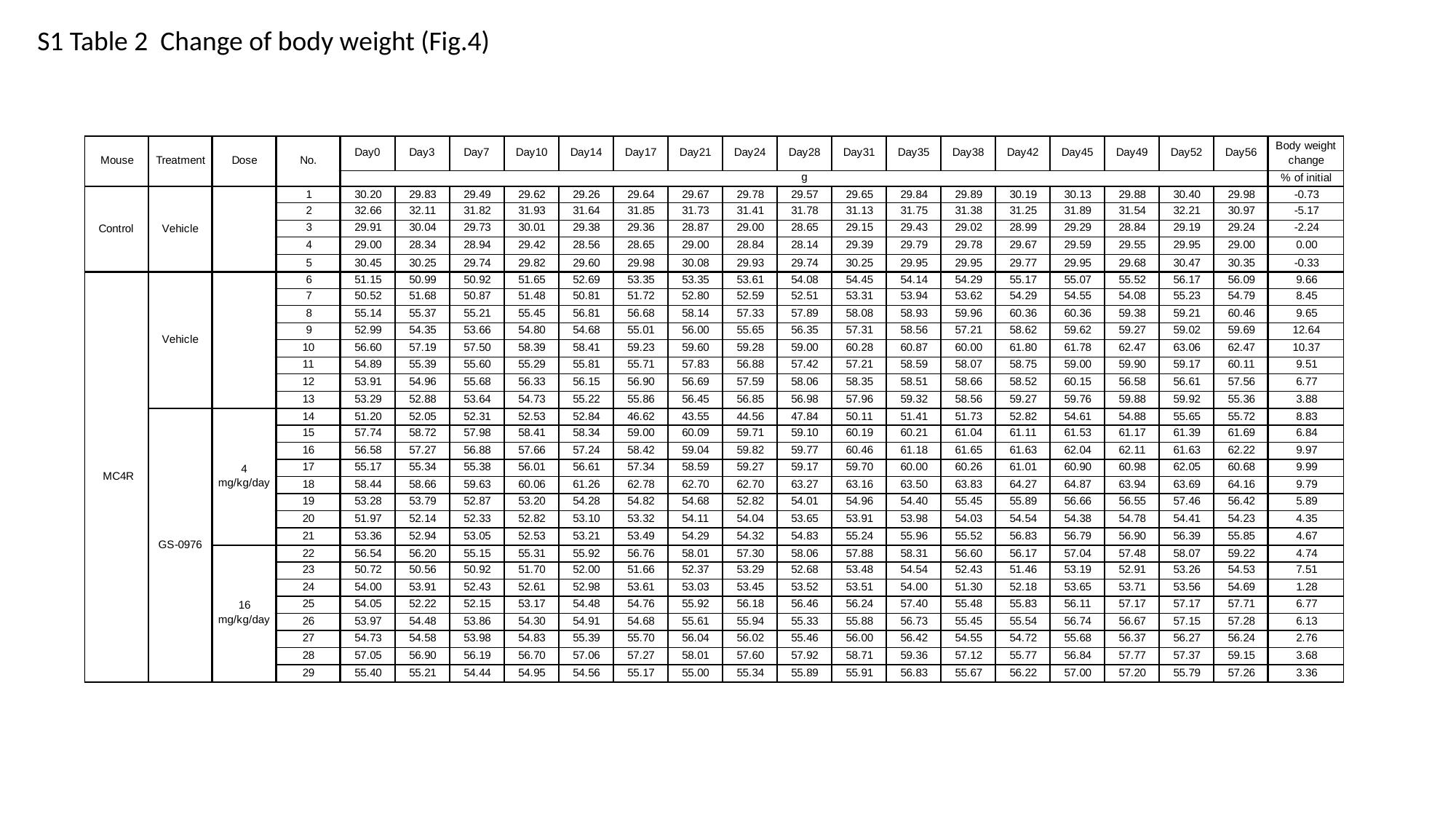

S1 Table 2 Change of body weight (Fig.4)

## Slide 3
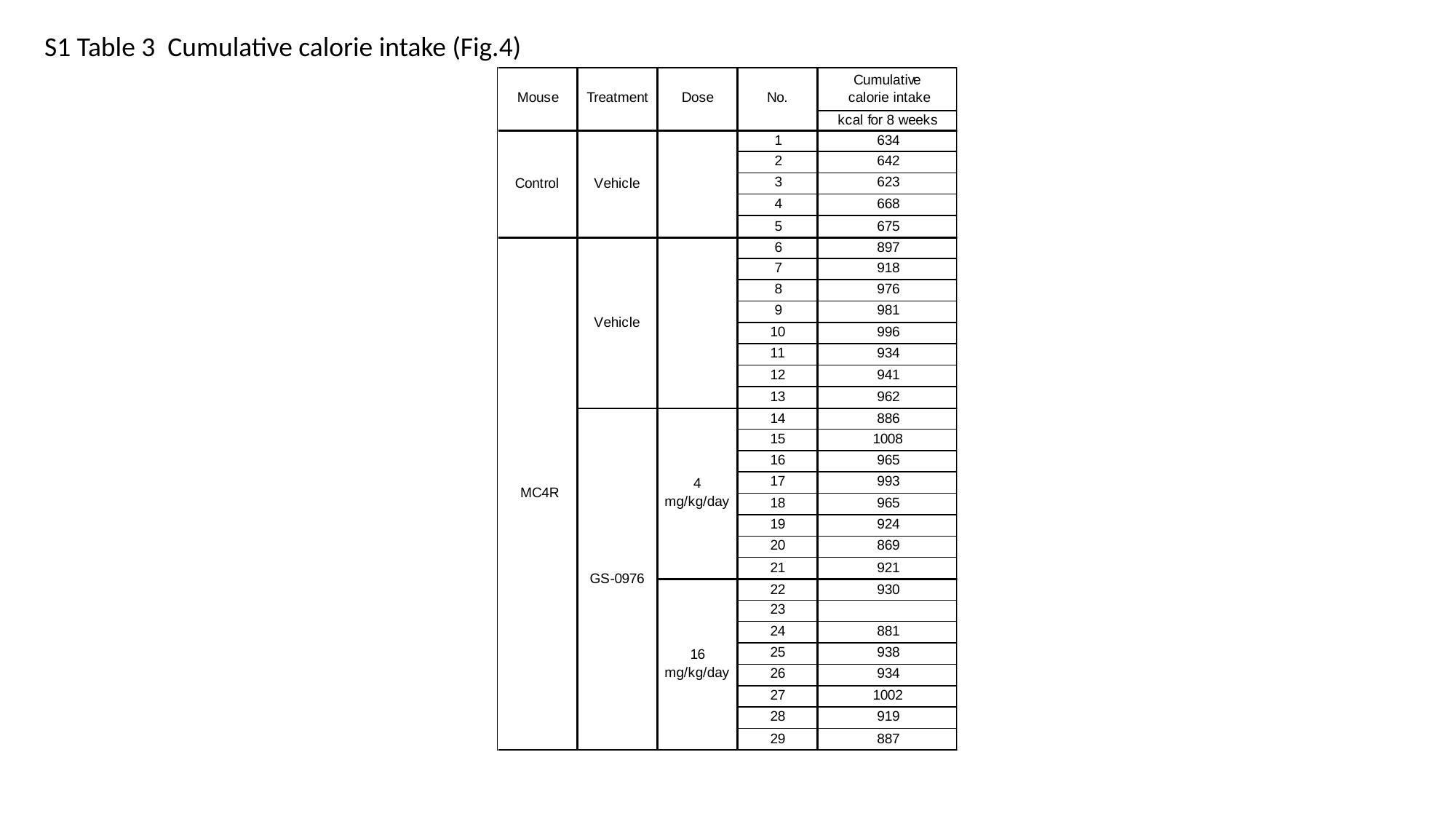

S1 Table 3 Cumulative calorie intake (Fig.4)

## Slide 4
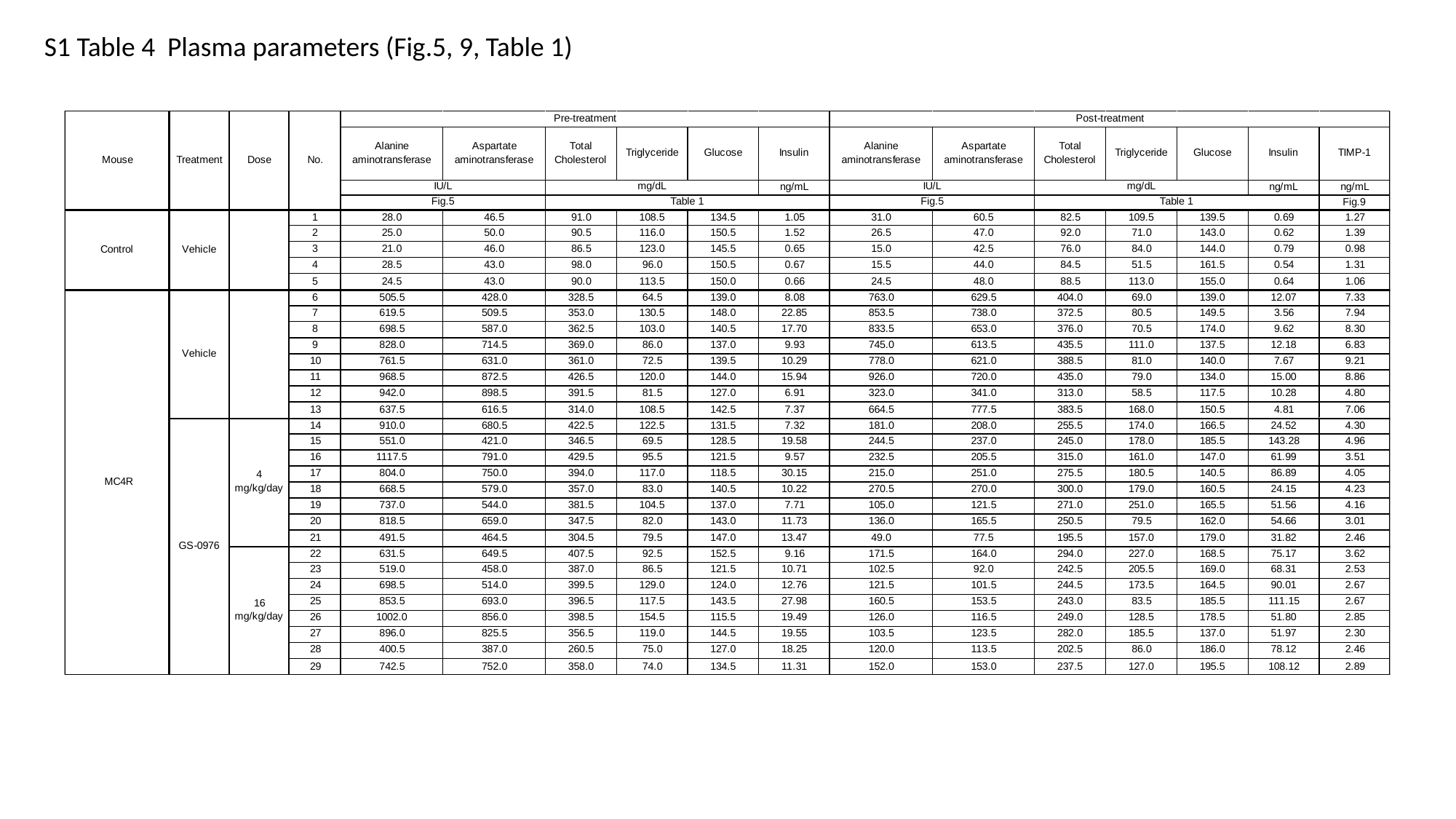

S1 Table 4 Plasma parameters (Fig.5, 9, Table 1)

## Slide 5
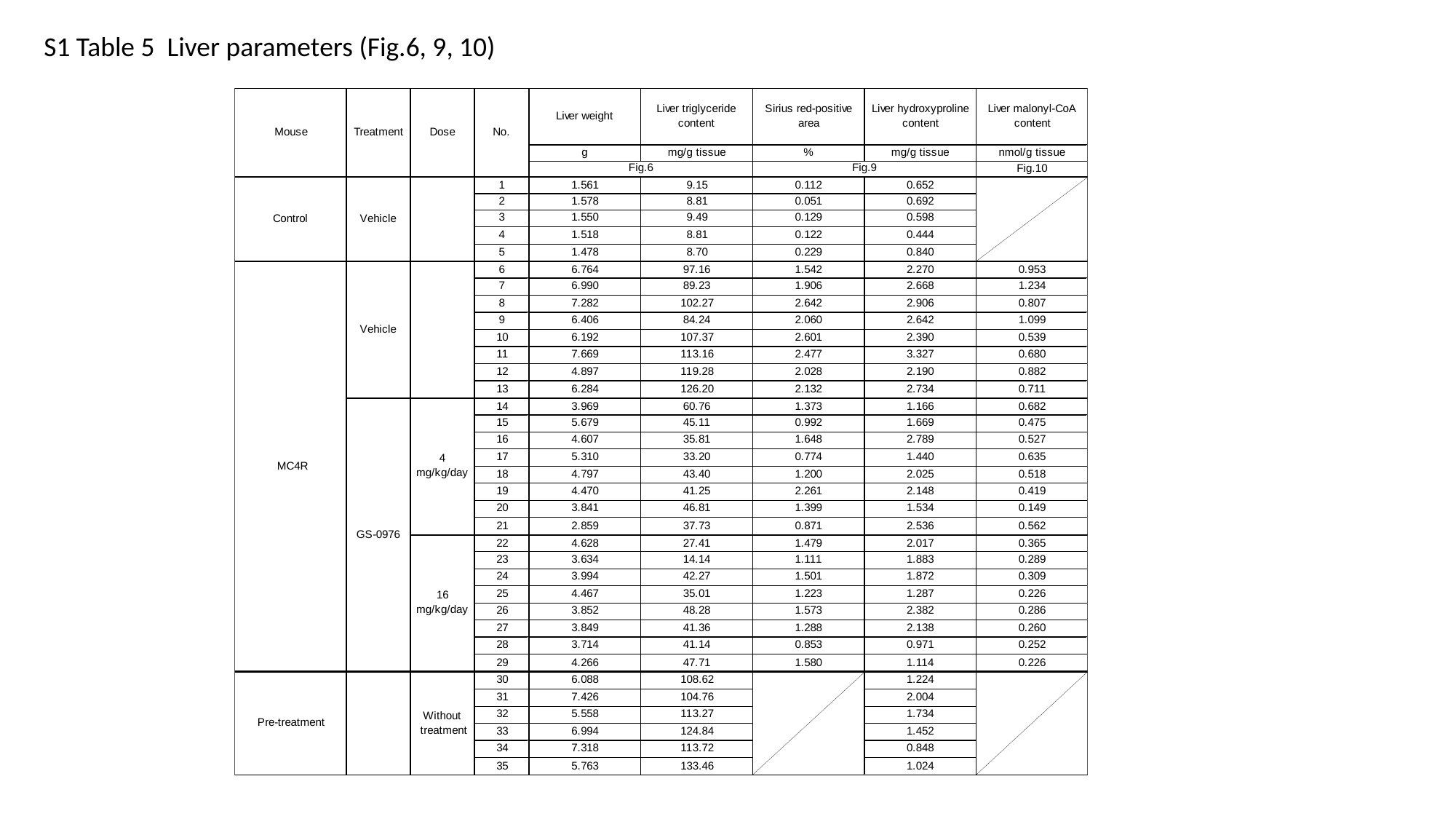

S1 Table 5 Liver parameters (Fig.6, 9, 10)

## Slide 6
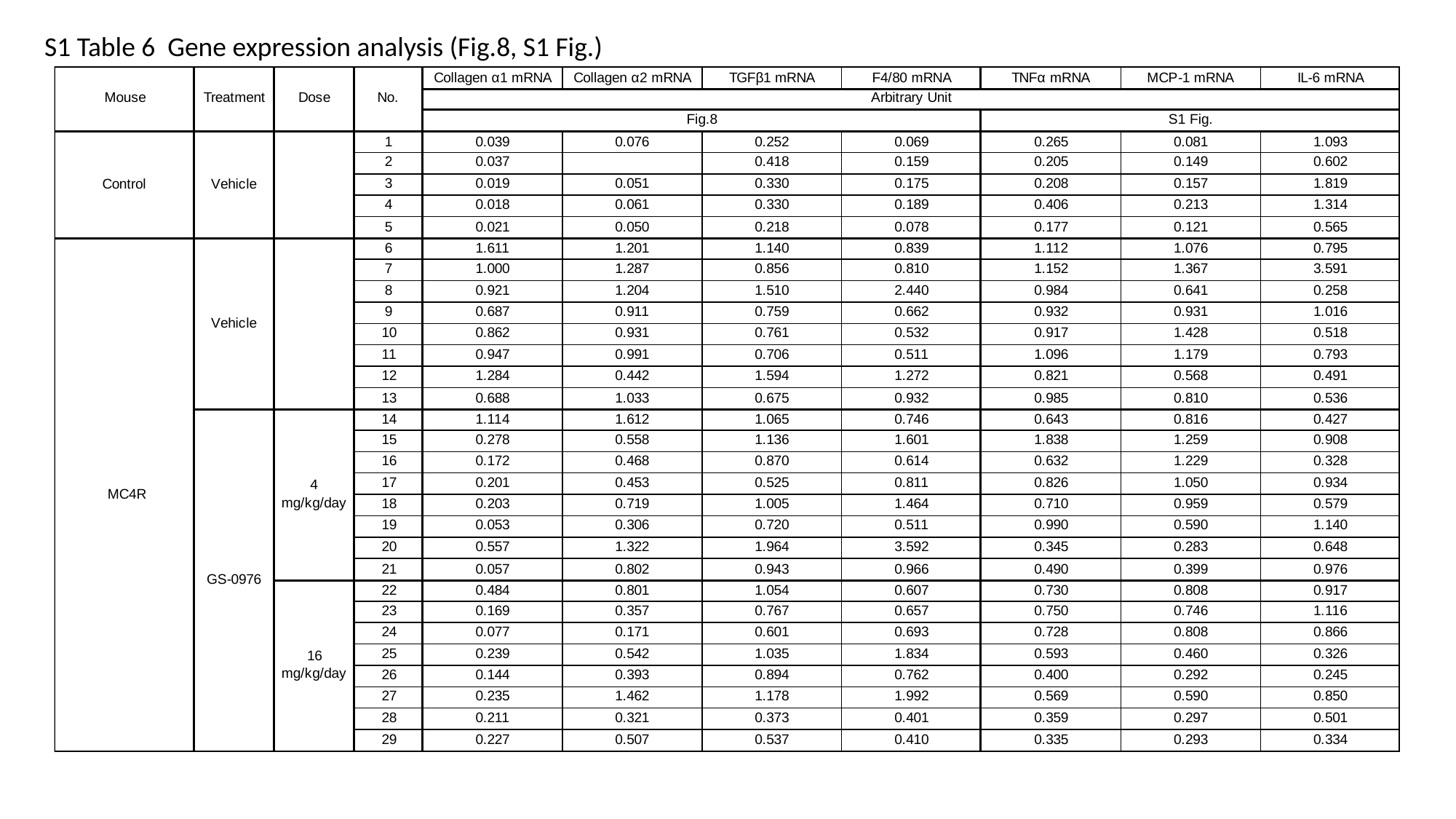

S1 Table 6 Gene expression analysis (Fig.8, S1 Fig.)

## Slide 7
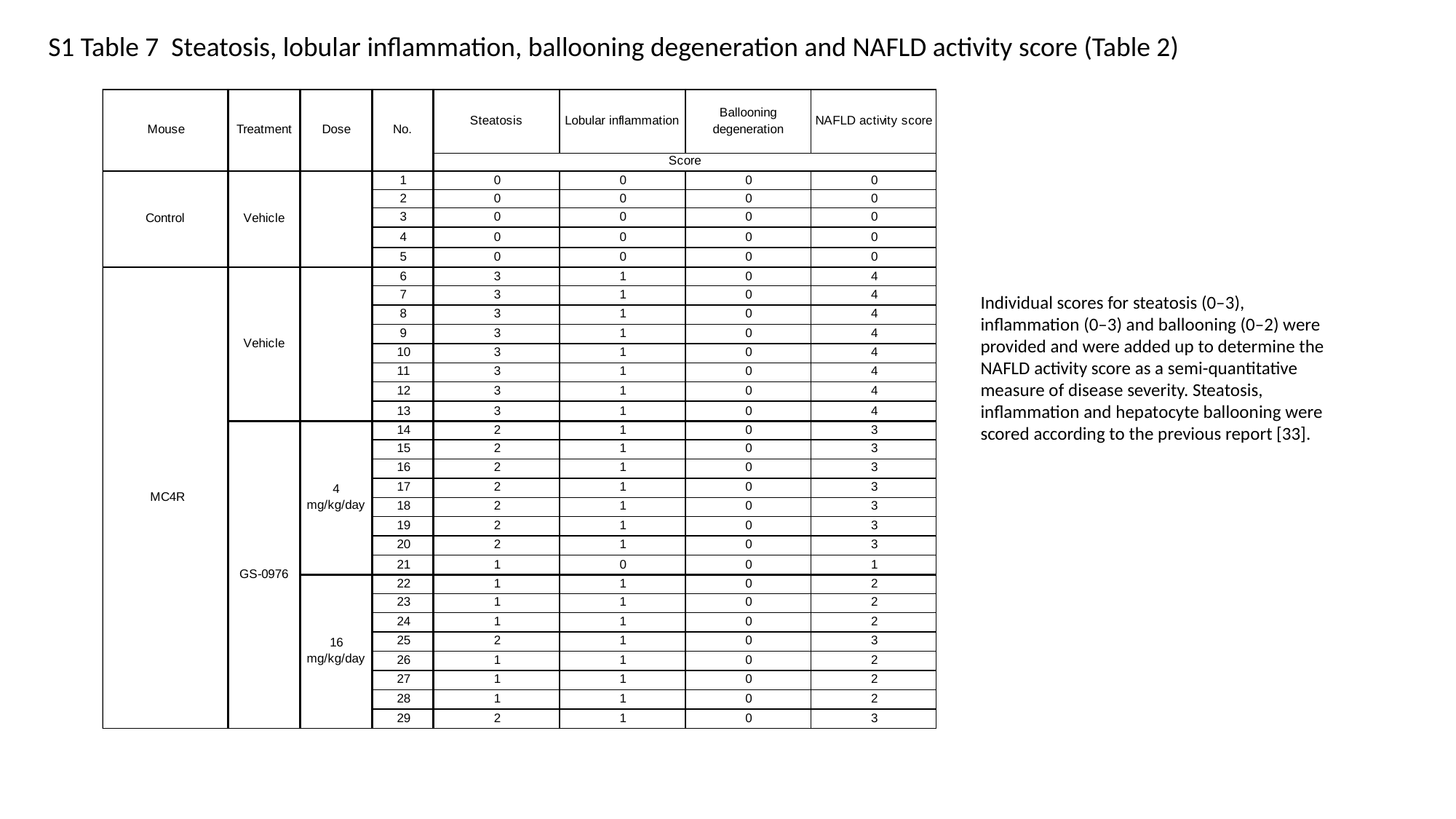

S1 Table 7 Steatosis, lobular inflammation, ballooning degeneration and NAFLD activity score (Table 2)
Individual scores for steatosis (0–3), inflammation (0–3) and ballooning (0–2) were provided and were added up to determine the NAFLD activity score as a semi-quantitative measure of disease severity. Steatosis, inflammation and hepatocyte ballooning were scored according to the previous report [33].
